# Multi-angle property analysis and stress–strain curve prediction of cementitious sand gravel based on triaxial test

**DOI:** 10.1038/s41598-024-62345-z

**Published:** 2024-07-16

**Authors:** Qingqing Tian, Lei Guo, Yiqing Zhang, Hang Gao, Zexuan Li

**Affiliations:** North China University of Water Resources and Electric Power, Zhengzhou, 450046 China

**Keywords:** Cementitious sand gravel, Cuckoo search, Damage analysis, Energy dissipation, Stress–strain curve, eXtreme Gradient Boosting, Failure characteristics, Civil engineering, Software

## Abstract

In order to further promote the application of cementitious sand gravel (CSG), the mechanical properties and variation rules of CSG material under triaxial test were studied. Considering the influence of fly ash content, water-binder ratio, sand rate and lateral confining pressure, 81 cylinder specimens were designed and made for conventional triaxial test, and the influence laws of stress–strain curve, failure pattern, elastic modulus, energy dissipation and damage evolution of specimens were analyzed. The results showed that the peak of stress–strain curve increased with the increase of confining pressure, and the peak stress, peak strain and energy dissipation all increased significantly, but the damage variable D decreased with the increase of confining pressure. Under triaxial compression, the specimen was basically sheared failure from the bonding surface, and the aggregate generally did not break. Sand rate had a significant effect on the peak stress of CSG, and decreased with the increase of sand rate. Under the conditions of the same cement content, fly ash content and confining pressure, the optimal water-binder ratio 1.2 existed when the sand rate was 0.2 and 0.3. After analyzing and processing the stress–strain curve of triaxial test, a Cuckoo Search-eXtreme Gradient Boosting (CS-XGBoost) curve prediction model was established, and the model was evaluated by evaluation indexes R^2^, RMSE and MAE. The average R^2^ of the XGBoost model based on initial parameters under 18 different output features was 0.8573, and the average R^2^ of the CS-XGBoost model was 0.9516, an increase of 10.10%. Moreover, the prediction curve was highly consistent with the test curve, indicating that the CS algorithm had significant advantages. The CS-XGBoost model could accurately predict the triaxial stress–strain curve of CSG.

## Introduction

In recent years, the country has placed significant emphasis on enhancing ecological construction, resource conservation, and environmental protection. This commitment aims to achieve carbon peak and carbon neutrality while prioritizing the enhancement of the ecological environment and the promotion of a green, low-carbon cycle^1,2^. Cementitious Sand Gravel (CSG) is a new type of dam construction material that is made by mixing cement material, water, bed gravel, excavation waste and other local materials with simple equipment^3,4^. Compared to standard concrete, CSG has a lower cement content, a higher water-cement ratio, and a larger aggregate size, which results in lower hydration heat, which speeds up construction. Due to the numerous advantages of the material, CSG is gradually becoming a very attractive alternative to traditional RCC in the field of dam construction^5–7^.

Relevant scholars have studied the uniaxial compressive, tensile and splitting properties of CSG. For example, Yang et al.^8^ conducted a uniaxial compression test and found that the compressive strength increased by 15–20% for every 10 kg/m^3^ increase in cement content. The economical and optimal amount of fly ash was 40% and 50% of the total cementing material (mainly cement and fly ash), respectively. Guo et al.^9^ obtained a linear relationship between the compressive strength and splitting strength of CSG cubes through strength tests, and the splitting strength was 7–12% of the compressive strength. Yang et al.^10^ studied the influence of cement content on the deformation characteristics of CSG materials, and the results showed that with the increase of cement content, the failure strain of CSG materials decreased, the brittleness increased, and the initial modulus increased exponentially. Due to the influence of material structural characteristics and external environmental factors, triaxial compression will have a significant impact on mechanical properties, so relevant scholars have also carried out multi-axial test research on CSG. For example, Lohani et al.^11^ and Kongsukprasert et al.^12^ analyzed the influence of mix design indexes such as curing age, water consumption, cement content and density on the shear strength of CSG materials through triaxial shear test. Haeri et al.^13–15^ analyzed the stress–strain characteristics of CSG materials under different cement content and found that the axial strain corresponding to the failure strength decreased with the increase of cement content, while the cohesion, strength and stiffness increased with the increase of cement content. Sun et al.^16^ successively carried out triaxial shear tests to analyze the stress–strain curve characteristics under different cement content. Wu et al.^17^ conducted triaxial shear tests on CSG materials and analyzed the effects of age on peak strength and stress–strain curve characteristics. However, the current research on CSG is still limited to a single force, and its triaxial mechanical properties under different factors are still to be explored. Although these studies on CSG properties have achieved some positive results, most of them are based on the analysis of test results of variable factors, which cannot reflect the potential law of CSG material properties from a statistical point of view, and lack of multi-factor correlation analysis based on data prediction.

Given the complexity, time consumption, cost, and susceptibility to external factors in the preparation process of CSG specimens, it is essential to explore alternative methods for acquiring stress–strain curves effectively. In previous studies, it can be found that many scholars tried to predict stress–strain curves of different materials. For example, Wang et al.^18^ described and predicted the stress–strain curves of loess by establishing mathematical models. Kasuya et al.^19^ conducted thermal simulation tests on steel and established a stress–strain curve prediction equation based on hardness with the test data. The results showed that as long as the strength of steel reaches 980 MPa, the error of the stress–strain curve predicted by the model was within 100 MPa in most cases. Xie et al.^20^ analyzed and predicted the variations of stress–strain curve of concrete under multi-factor coupling by using parametric equation method. However, the general steps such as the establishment of mathematical models or mathematical equations are complicated, and the most important thing is that the efficiency and accuracy need to be improved, so it is urgent to develop a low-cost, high-accuracy stress–strain curve prediction method. However, similar to the establishment of mathematical models or mathematical equations, the general steps are cumbersome, and more importantly, the efficiency and accuracy need to be improved. Therefore, it is urgent to develop a low-cost, high-accuracy stress–strain curve prediction method.

Machine learning, the cornerstone of artificial intelligence, is currently in a flourishing era of accelerated growth and advancement. The eXtreme Gradient Boosting (XGBoost) algorithm, as one of the major machine learning algorithms, stands out as an efficient and rapid ensemble learning tool. It has found extensive practical applications in data mining across diverse domains such as finance^21,22^, hydrology^23,24^, materials^25,26^, and automation^27,28^ and other fields of data mining. XGBoost has consistently showcased outstanding learning performance and classification accuracy. However, XGBoost and other machine learning models all have hyperparameter optimization problems, that is, the generalization ability of the same input data built by using different hyperparameters is different^29,30^. Grid search^31–33^, Bayesian optimization^34,35^ and random search^36,37^ are all feasible methods to find the optimal hyperparameter. However, when the hyperparameter dimension is too high, the calculation cost of grid search is high, and it is difficult to guarantee to find the optimal hyperparameter combination. After a long period of development, there have been many researches on coupled artificial intelligence algorithms and swarm intelligence optimization methods, such as ant colony algorithm^38,39^, whale algorithm^40,41^, fruit fly algorithm^42,43^, etc., which are used for parameter optimization, but the algorithm's own parameter setting and the applicability of threshold setting make it difficult to ensure that the optimization results reach the global optimal. Cuckoo Search (CS) algorithm has strong global search ability and global convergence. It is proved that the algorithm has significant advantages in accuracy, complexity and stability by constructing a Markov model that follows the Levy distribution according to the convergence algorithm criteria^44^. Furthermore, this algorithm possesses fewer parameters, facilitating seamless integration with other artificial intelligence algorithms. Its robustness is high, allowing for a broader range of applications^45^.

The research on the performance of CSG was mostly focused on the uniaxial compression, tension, splitting, etc., which was limited to a single stress state. In order to further reveal the mechanical properties and variation rules of CSG materials under triaxial stress state, this paper took CSG as the research object and considered the influence of four variation parameters, such as fly ash content, water-binder ratio, sand rate and lateral confining pressure value. A total of 81 cylindrical specimens were designed and fabricated for conventional triaxial tests to determine the corresponding stress–strain relationship curves, peak stress with confining pressure, confining pressure with strain values, and failure pattern of CSG under different combinations and proportions. Simultaneously, further research was conducted on CSG through analysis of their elastic modulus, energy dissipation, and damage.

Furthermore, given the pressing demand for a precise and efficient prediction method for the triaxial stress–strain curve of CSG, along with the benefits offered by the XGBoost algorithm and the CS optimization algorithm. Based on the CS optimization theory, this paper analyzed and processed the data based on the stress–strain curve data obtained from the test. For the first time, an XGBoost model based on CS (CS-XGBoost) was constructed to achieve accurate prediction of the CSG triaxial stress–strain curve, which provided reference for the theoretical research and engineering application of CSG.

## Experiment design and method

### Experiment raw materials and mix ratio

The raw materials utilized in this test mainly comprised natural pebbles, cement, fly ash, natural river sand, and water. The coarse aggregate used was natural cobblestone, which was mainly sourced from the project site. The fine aggregate was sand material, purchased from Tanghe Xinmiao Sand and Gravel Co., LTD., with fineness modulus of 2.94, which was medium sand. The cement was grade P·O42.5 cement purchased from Tianrui Cement Group Co., LTD., and its physical and mechanical properties were shown in Table 1; the fly ash was purchased from Yulian Power Plant, and its technical properties were shown in Table 2. Relevant researchers had conducted many studies on CSG with a cement content of 50 kg/m^3^. For example, Huang et al.^46^ and Chen et al.^47^ conducted relevant performance tests and analyses of CSG with a cement content of 50 kg/m^3^, so the cement content of this triaxial test was 50 kg/m^3^. According to the *Technical Guidelines for Dam Construction with Cemented Granular Materials (SL 678-2014)*, the amount of cementing material (total amount of cement and fly ash) should not be less than 80 kg/m^3^, so the content of fly ash should not be less than 30 kg/m^3^. Combined with the relevant conclusions of the *Study on Ultra-Poor Cemented Material DAMS*, according to the common ratio of projects, the total amount of cementing material was controlled under 80 kg/m^3^, 90 kg/m^3^ and 100 kg/m^3^, so the amount of fly ash used in this test was 30 kg/m^3^, 40 kg/m^3^ and 50 kg/m^3^ respectively. According to the *Technical Guidelines for Dam Construction of Cemented Granular Materials* (*SL 678-2014*) and the original test results, the optimal water-binder ratio of CSG materials ranges from 0.7 to 1.3. In practical projects, taking Shoukou Fortress, which is currently under construction in China, as an example, the water-binder ratio used was 1.58. The influence of large water-binder ratio on the mechanical properties of CSG materials should also be included in the research scope, so the optimal water-binder ratio range was set as the middle value and the upward value, and the water-binder ratio values were set as 1.0, 1.2 and 1.4 in this test. The size of the sand rate will affect the compactness of the specimen and the cementation of the material, as well as the amount of cement, thus affecting the cost of the material. Taking the above factors into account and referring to the sand rate involved in the *Study of Ultra-Poor cementing Material Dam*, the sand rate in this test was 20%, 30% and 40%, respectively. A total of 27 groups cooperated in this experiment, as shown in Table 3.

**Table 1:** Physical and mechanical properties of cement.

Cement type: Ordinary portland cement Strength class: 42.5					
Technical requirement	Standard value	Test value	Technical requirement (MPa)	Standard value	Test value
Stability	Qualified	Qualified	3d flexural strength	≥ 3.5	5.2
SO_3_ (%)	3.5	2.60	Monolithic strength		
MgO (%)	5.0	–	3d Compressive strength	≥ 17.0	28.3
Ignition loss (%)	≤ 5.0	3.03	Monolithic strength		
Initial setting time (min)	≥ 45	168	28d flexural strength	≥ 6.5	–
Final setting time (min)	≤ 600	230	Monolithic strength		
Cl^-^ (%)	≤ 0.06	0.025	28d Compressive strength	≥ 42.5	–
Alkali content (%)	–	–	Monolithic strength

**Table 2:** Technical properties of fly ash.

Density (g/cm^3^)	45 μm residual (%)	Water demand ratio (%)	Chemical composition (%)				
			SiO_2_	Fe_2_O_3_	Al_2_O_3_	CaO	Ignition loss
2.11	17	102	59.61	7.41	21.33	4.24	1.78

**Table 3:** Mix proportion.

Group	CF31	CF32	CF33	CF34	CF35	CF36	CF37	CF38	CF39	CF41	CF42	CF43	CF44	CF45
	CF46	CF47	CF48	CF49	CF51	CF52	CF53	CF54	CF55	CF56	CF57	CF58	CF59	-
Fly ash (kg/m^3^)	30	30	30	30	30	30	30	30	30	40	40	40	40	40
	40	40	40	40	50	50	50	50	50	50	50	50	50	–
Water-binder ratio	1.0	1.0	1.0	1.2	1.2	1.2	1.4	1.4	1.4	1.0	1.0	1.0	1.2	1.2
	1.2	1.4	1.4	1.4	1.0	1.0	1.0	1.2	1.2	1.2	1.4	1.4	1.4	–
Sand ratio	0.2	0.3	0.4	0.2	0.3	0.4	0.2	0.3	0.4	0.2	0.3	0.4	0.2	0.3
	0.4	0.2	0.3	0.4	0.2	0.3	0.4	0.2	0.3	0.4	0.2	0.3	0.4	–

### Specimen design and preparation

**Figure 1:**
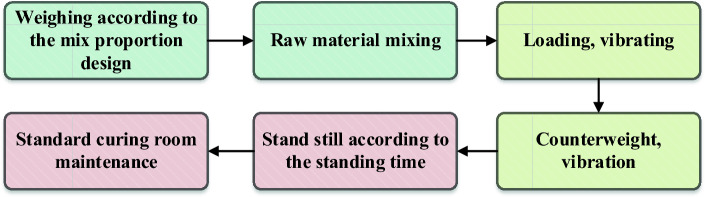
Production process of CSG specimen.

The test was carried out according to the *Technical Guidelines for Dam Construction with Cemented Granular Materials* (*SL 678-2014*), *Test Regulations for hydraulic Concrete* (*SL 352-2006*), and *Standard of Test method for long-term performance and Durability of ordinary Concrete* (*GB/T 50082-2009*). The specimen size was Φ150mm × H300mm cylinder, the production process is shown in Fig. 1: Material preparation: Weighed according to the calculated amount of material; Material mixing: Before mixing, according to the test mix ratio, weighed the cementing material and aggregate into the mixer in turn, stirred for 10 s first, so that the mix was fully turned and evenly mixed, then added water with accurate weighing, covered the drum cover of the mixer, and stirred for 2 min; Material loading: Before loading the mold, the inner wall of the test mold was evenly coated with oil, and then the mixed CSG material was divided into two layers into the test mold that had been smeared with mineral oil, each layer of material thickness was roughly equal. The method of manual vibration was used during loading, and the number of interpolations was not less than 25 times. At the same time, the specimen needed to be loaded twice with pressure vibration molding; Vibration compaction: This test adopted roller compacted concrete forming method, using magnetic shaking table (with magnetic shaking table, can fix the iron test mold, so that it does not shake back and forth, to prevent uneven amplitude) to press down vibration forming; Specimen maintenance: After molding, the specimens were placed at 20 ± 5 °C indoors for 24–48 h to be solidified. After standing for a period of time, the mold was removed and numbered. Subsequently, the specimens were immediately moved to a curing room (with a temperature controlled at 205 ± 25 °C and a relative humidity of over 95%) for curing. Placed the test piece son the support, maintained a certain distance between the test pieces, and kept the surface of the test piece moist.

### Triaxial test

**Figure 2:**
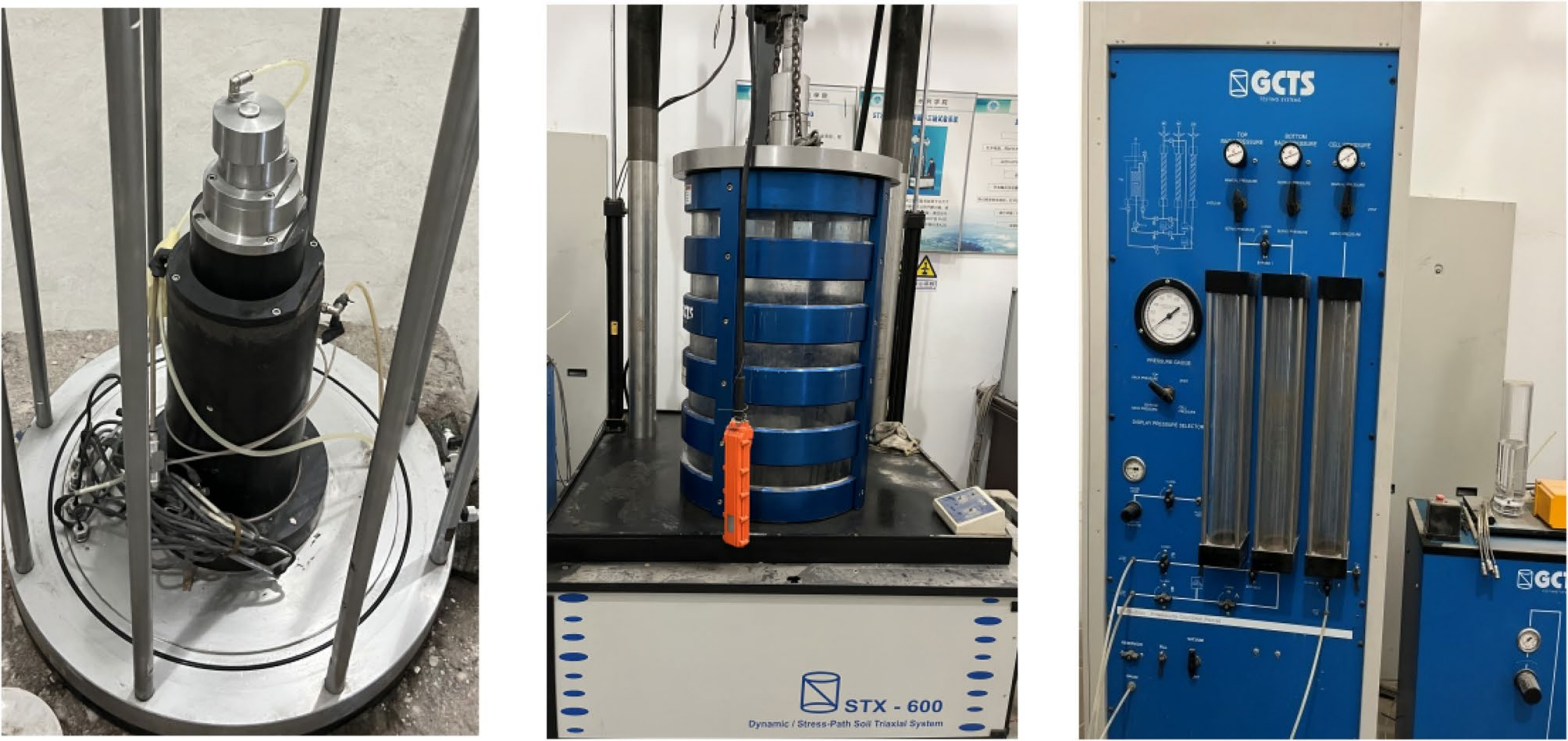
Triaxial test system of CSG specimen in this paper.

The STX-600 large-scale dynamic triaxial test system of American GCTS company was adopted in the test, as shown in Fig. 2. The test machine is composed of four parts: loading system, deformation measurement system, load and displacement control system, and data acquisition system. The STX-600 triaxial test system is used for dynamic testing of large particle size soils such as gravel and railway ballasts. The three-axis system accepts samples up to 300 mm in diameter and up to 700 mm in height. The system is commonly used to perform liquefaction, elastic modulus, cyclic strength, complex modulus, and other dynamic triaxial tests, including synchronous cyclic axial and confining stress loading. The system also provides the necessary versatility to automatically perform traditional three-axis tests as well as advanced procedures such as stress or strain paths.

The experiment was unconsolidated and undrained. For large gravity DAMS or arch DAMS and other projects, the confining pressure level can reach 600 kPa or above. CSG is a new type of building material, and 400–800 kPa already includes confining pressure levels corresponding to most conventional DAMS, so the confining pressure of the test was selected as 400 kPa, 600 kPa and 800 kPa. The mechanical response of materials under different confining pressure conditions could be deeply understood, which was helpful to understand the stress–strain characteristics and mechanical properties of materials^48–50^. Strain control was adopted during the test, and the shear rate was 0.2%/min. The specific operation steps were as follows: Loading sample and sealing pressure chamber: Wrapped the specimen with rubber film, placed it on the base, lowered the pressure chamber and sealed it; Pressurization: The confining pressure was selected by the computer for both input and feedback adjustment, and then the surrounding pressure was applied to the specimen step by step. During each test, a fixed confining pressure was applied to the specimen until the confining pressure reading on the computer was equal to the confining pressure value set by the input and stable; Setting parameters: After the confining pressure was set, the Test option on the toolbar of CATS software was opened and the parameters of the sample were set in turn; Naming and execution: the test samples were numbered and named successively, and the corresponding parameters of the samples were input to execute the program; Data output: Output in Excel text format.

### Ethical statement

No experiments on animals and humans are conducted in this article. There are no human subjects in this study and informed consent is not applicable.

## Test results and analysis

### Triaxial stress–strain curve analysis

**Figure 3:**
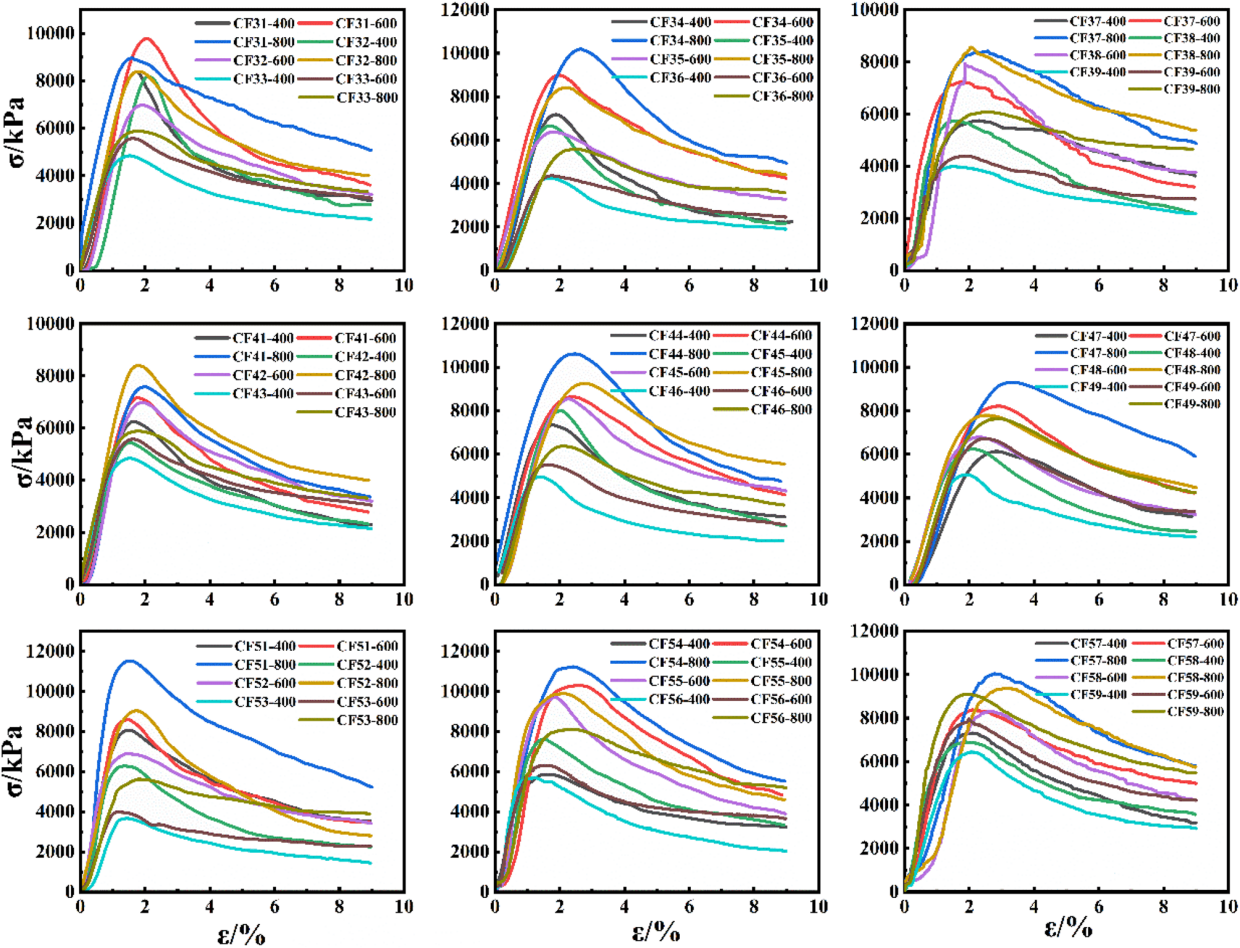
Stress–strain curve results of triaxial tests of different CSG specimens.

The stress–strain curves of specimens with varying parameters were sorted out as shown in Fig. 3. It can be seen from Fig. 3 that the CSG material exhibited linear elastic properties at low stress levels, and entered the plastic stage with the gradual increase of stress until it reached the peak strength. Eventually it approached residual strength. In general, the stress–strain curve could be divided into three stages:

(1) Approximate line segment. From the beginning of loading to the stress reached about 75% of the ultimate strength of CSG material, the stress of the specimen increased linearly with the increase of strain, and the elastic ultimate strength was reached when the strain was about 1%. When the strain ranged from 0 to 1%, the CSG materials exhibited obvious linear elastic characteristics. (2) Curve rising segment. As the strain continues to increase, when the stress state of the material reached the limit and the specimen could not bear the load due to serious damage and cracking, the ultimate strength reached by the material was called the peak strength, and the corresponding strain value was called the peak strain. (3) Curve descent section. After the peak strength was exceeded, the specimen cracked seriously, so that the material was in a state of unstable expansion, and its ability to bear the load decreased.

As could be seen from Fig. 3, the stress–strain curve was greatly influenced by fly ash content, sand rate and lateral confining pressure value, while the water-binder ratio had relatively little influence. With the gradual increase of the lateral confining pressure value, the circumferential constraint effect was strengthened, and the bearing capacity of the specimen was gradually increased, so the peak point of the curve was gradually increased.

Sand rate was an important factor affecting the material strength of CSG. As could be seen from Fig. 3, under the same cement content, fly ash content, water-binder ratio and confining pressure, the peak stress–strain curves with sand rate of 20%, 30% and 40% respectively showed an obvious decreasing trend. At the same time, when the sand rate was 20%, the peak stress–strain curve was significantly higher than that when the sand rate was 30% and 40%, which was similar to the maximum stress of the CSG material mentioned in the previous study when the sand rate was 20^51^. The reason for the analysis was that, unlike other concrete materials, CSG material was mainly composed of cementified material, sand and gravel and water, which were simply mixed to wrap aggregate and thus form a certain strength. With the increase of sand rate, the amount of cementified material pulp on the surface of the aggregate was relatively small when the amount of cementified material was certain, which resulted in a relative decline in the cementing force between aggregates. The workability of the mixture was also poor. Secondly, due to the different particle sizes of aggregate of CSG material, holes would be formed inside the specimen. With the increase of sand rate, these holes would be gradually filled with sand. However, due to the small cementing force between sand particles, poor bearing capacity and instability, failure surfaces would be easily formed under external load and rapid failure would result in the reduction of material strength.

The incorporation of fly ash into CSG not only improved the strength of the material, but also effectively improved the durability of the material^52^. As can be seen from Fig. 3, under the same cement content, sand rate, water-binder ratio and confining pressure, the peak stress–strain curve with fly ash content of 50 kg/m^3^ was significantly greater than that with fly ash content of 30 kg/m^3^ and 40 kg/m^3^. However, when fly ash content was 30 kg/m^3^ and 40 kg/m^3^, the peak stress–strain curves had little difference.

The influence of water consumption on the strength of CSG was particularly important. As could be seen from Fig. 3, under the same cement content, fly ash content and confining pressure, the peak stress–strain curve of water-binder ratio 1.2 at sand rate of 0.2 and 0.3 was significantly greater than water-binder ratio 1.0 and 1.4, and there was an inflection point, that was, there was an optimal water-binder ratio 1.2. However, when the sand rate was 0.4, the peak value of the force-strain curve of water-binder ratio 1.0, 1.2 and 1.4 was not much different, but the peak value of the stress–strain curve was the lowest when the water-binder ratio is 1.0.

### Feature point parameter

#### Confining pressure and peak stress

**Figure 4:**
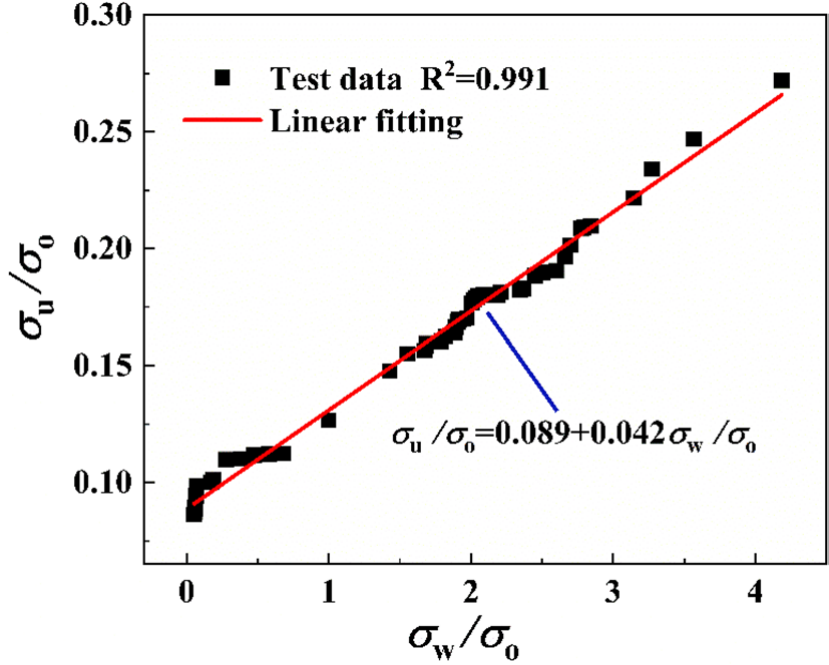
Fitting curves of confining pressure and peak stress.

Figure 4 showed the change of peak stress of CSG material under different lateral confining pressures. It could be seen that the confining pressure had a significant effect on the peak stress, and the peak stress increased linearly with the increase of the confining pressure. By fitting the test data, the calculation formula of the peak stress and confining pressure value of CSG material was obtained:

1 $$\frac{{\sigma_{{\text{u}}} }}{{\sigma_{{\text{O}}} }} = 0.089 + 0.042\frac{{\sigma_{{\text{w}}} }}{{\sigma_{{\text{O}}} }},R^{2} = 0.991$$

where *σ*_u_ is the corresponding peak axial stress; *σ*_0_ is the peak stress of the specimen under uniaxial compression.

In addition, the larger the slope of the fitted curve in Fig. 4, the greater the peak stress of the CSG under the same confining pressure value. Combined with the experimental phenomenon of the total failure of aggregate in the section of CSG materials under triaxial compression, it could be seen that since CSG materials were different from concrete materials, aggregates were mainly cemented together through cementitious materials. Under the condition of failure load, intergranular cementitious materials were the first to be destroyed, and aggregates would not be broken. Therefore, the strength of cementitious materials became an important factor restricting the peak stress of CSG materials under triaxial compression, and the greater the confining pressure value, the more obvious the strength defect of CSG materials.

**Figure 5:**
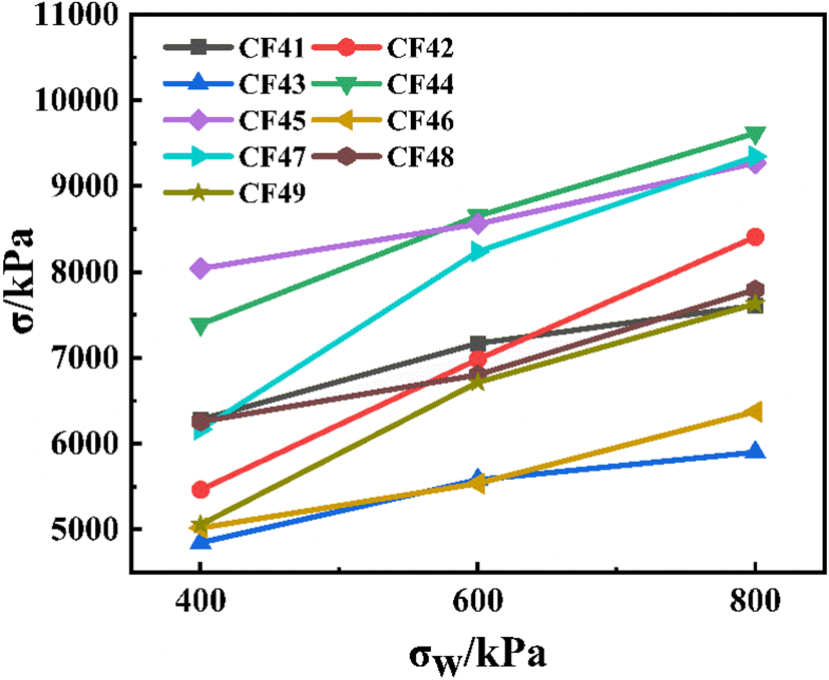
Confining pressure and peak stress.

In order to further intuitively analyzed the relationship between confining pressure and peak stress, CF41-49 was taken as an example to draw the confining pressure and peak stress, as shown in Fig. 5. It could be seen that the peak stress increased with the increase of confining pressure value, but the growth rate of peak stress slowed down with the increase of confining pressure value. On the one hand, because the lateral confining pressure was relatively small, the confining pressure had a significant reinforcing effect on weak areas such as the aggregate bonding interface and cement-based primary cracks inside the specimen. However, with the increase of confining pressure value, micro-cracks inside the concrete began to increase with the gradual increase of specimen deformation, resulting in a slowdown in the growth rate of peak stress. On the other hand, with the increased of confining pressure value, the triaxial stress of the specimen under the state of hydrostatic pressure would be larger, resulting in the accumulation of damage of the specimen before formal loading. The results were consistent with the above described rules.

#### Confining pressure and peak strain

**Figure 6:**
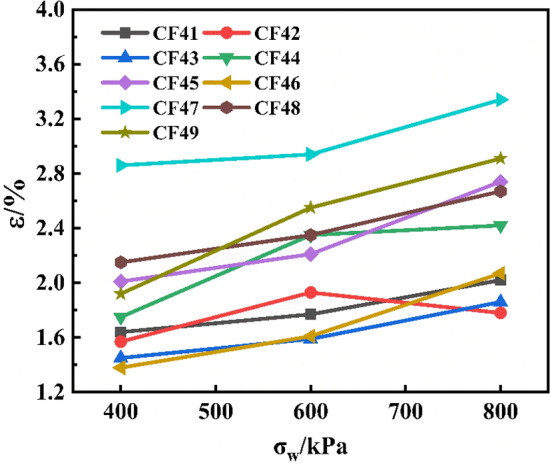
Confining pressure and peak strain.

Also taking CF41-49 as an example, the confining pressure and peak strain were plotted as shown in Fig. 6. It could be seen that the corresponding strain value when the CSG material reached the peak failure value was about 2%, and the strain value also increased with the increase of the confining pressure value. The reason for the analysis was that the main aggregate of the CSG material was pebble, and the inside of the test pieces were mostly porous structures. Under the condition of low confining pressure, the compactness between the test pieces was low, and the ductility was poor. With the increase of confining pressure, the specimen was subjected to greater external load force, the closer the aggregate was compacted, the better the overall compactness and ductility of the specimen, and the greater the change of axial displacement during failure. Lateral confining pressure was the main factor affecting the peak strain of the specimen, and the peak strain of the specimen increased gradually with the increase of confining pressure value. On the one hand, the increase of peak strain was due to the constraint effect of lateral confining pressure, which increased the elastic interval of the specimen. On the other hand, the increase of lateral confining pressure also gradually increased the plastic deformation of the specimen at the peak strain due to the extrusion flow of cement mortar and the development of fine cracks.

### Failure pattern analysis

**Figure 7:**
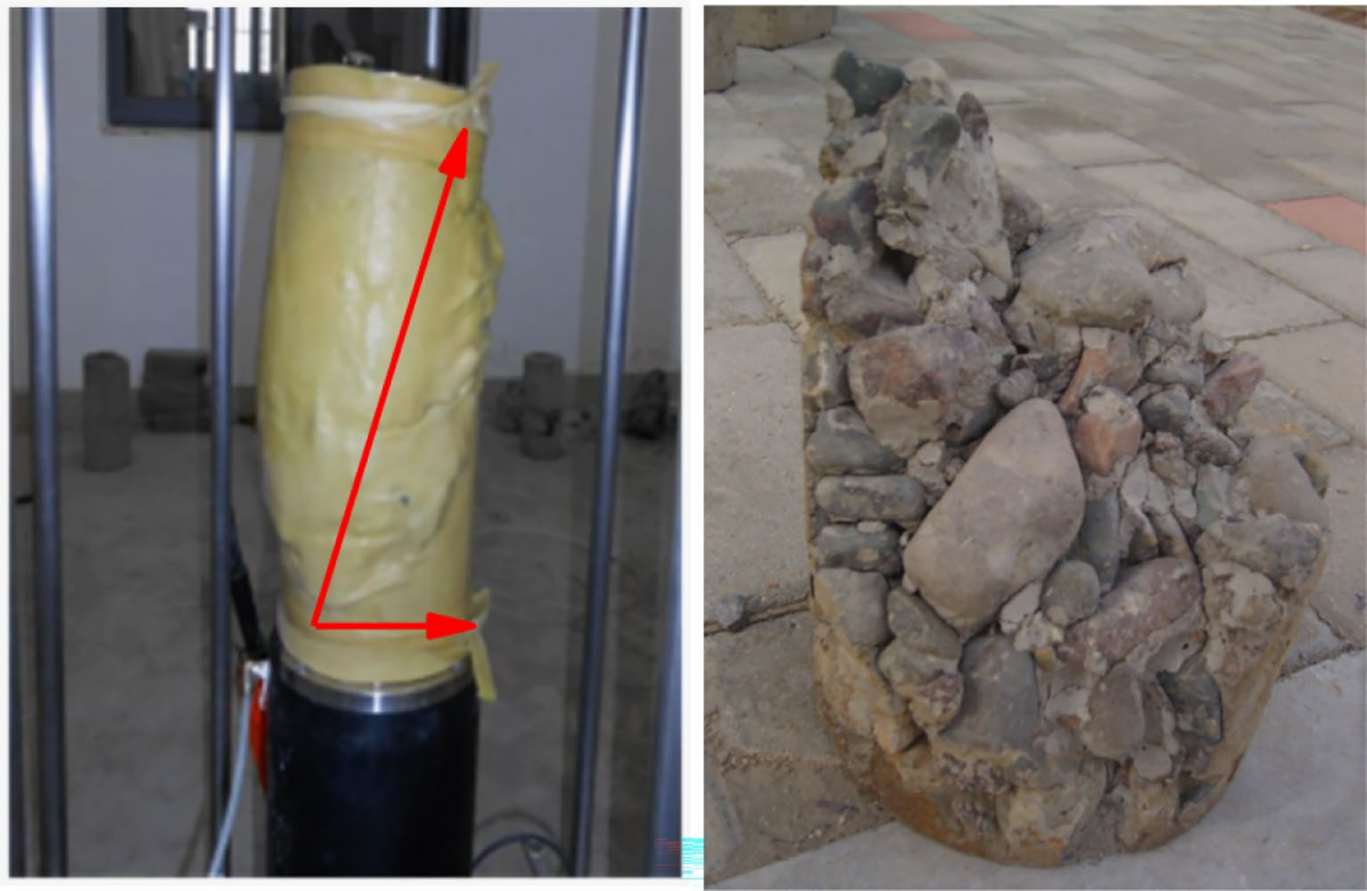
Failure pattern of CSG specimen.

In the process of shear failure, the specimen expanded to the periphery, showing obvious dilatancy characteristics. After shear, some specimens showed relatively obvious shear failure plane, as shown in Fig. 7. However, the shear failure plane of some specimens was not obvious after failure, or it was in the state of loose grain. According to the analysis of the reasons, the CSG material had obvious brittle failure characteristics, which was because under the combined action of normal stress and shear stress, the dislocation and extrusion between the particles of the test block tended to move in a certain direction, and finally formed a weak shear plane, along which the sample was destroyed. However, when the sand rate was large, the filling material between the aggregate particles inside the specimen was mainly sand. After the shear failure of the specimen, the dislocation arrangement between the aggregate particles was irregular, and the shear failure surface formed was not fixed, and the specimen became loose particles after the failure. The specimen was basically shear failure from the cementation surface, and the aggregate generally did not break. The failure of the whole specimen was mainly caused by cementation failure, which could also be verified by laboratory tests. The aggregate was not destroyed, but the CSG material had been separated from the aggregate, so the strength of the sample was determined by the amount of CSG added and the degree of coating. Analyzing the reasons, CSG materials were different from concrete materials. They mainly bonded the aggregates together through cementitious materials. Under failure loads, the first thing to be damaged was the inter particle cementitious material, which would not break the aggregates.

### Modulus of elasticity

**Figure 8:**
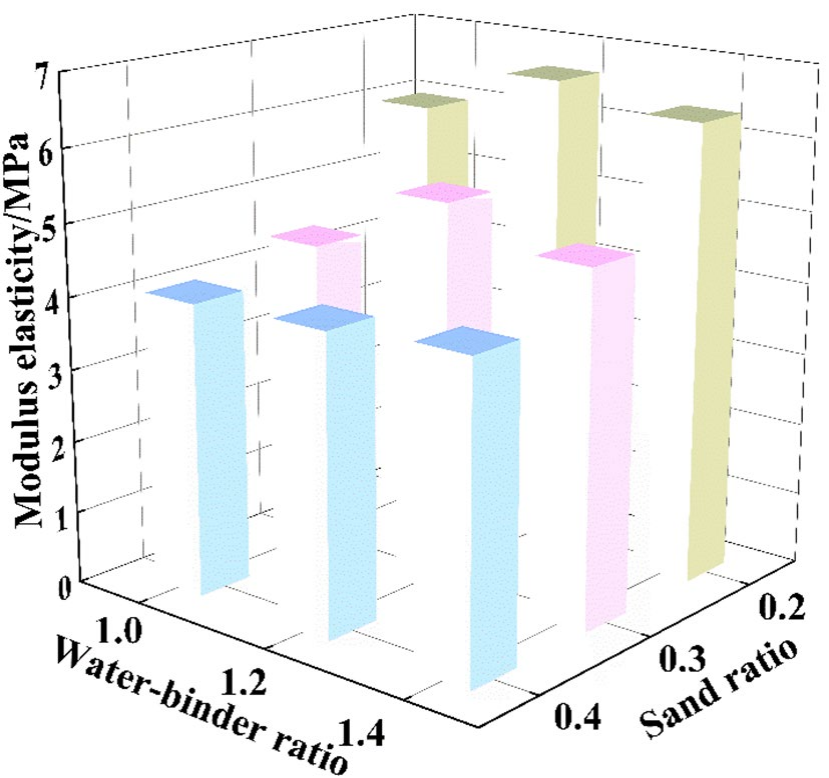
Elastic modulus and sand rate.

According to the stress–strain curve obtained by triaxial test, it could be seen that the stress of the material sample increased linearly with the increase of strain at low stress level, and the material exhibited linear elastic properties in the early stage. In this stage, the slope of the curve was the initial elastic modulus. Taking the fly ash content of 40 kg/m^3^ and the confining pressure of 800 kPa as an example, the calculation results were shown in Fig. 8. It could be seen that with the increase of sand rate, the elastic modulus of CSG decreased nonlinear, and with the increase of sand rate, the elastic modulus of CSG decreased gradually. The reason was that, on the one hand, the CSG material had obvious brittle failure characteristics, which was because under the combined action of normal stress and shear stress, the dislocation and extrusion between the particles of the test block tended to move in a certain direction, and finally formed a weak shear plane, along which the sample was destroyed. On the other hand, when the sand rate was large, the filling material between the aggregate particles inside the specimen was mainly sand, and the internal pores were easy to be compressed and deformed, resulting in the reduction of elastic modulus.

The water-binder ratio was the main factor affecting the compressive strength and compressive elastic modulus of CSG materials. When the water-binder ratio increased, the compressive strength and compressive elastic modulus of CSG materials also increased and reached a peak value, and then when the water-binder ratio increased again, the compressive strength and compressive elastic modulus of CSG materials decreased, that was, there was an “optimal water-binder ratio”. When fly ash was 40 kg/m^3^, confining pressure was 800 kPa and sand rate was 0.2 and 0.3, the elastic modulus of water-binder ratio 1.2 was significantly greater than that of water-binder ratio 1.0 and 1.4. An inflection point occurred, that was, there was an optimal water-binder ratio of 1.2. However, when the sand rate was 0.4, the peak stress–strain curves of water-binder ratio 1.0, 1.2 and 1.4 had little difference, but the elastic modulus was the lowest when the water-binder ratio is 1.0. The calculation of other mix ratios was also the same, which was consistent with the rule of 3.1 research results.

### Energy dissipation

**Figure 9:**
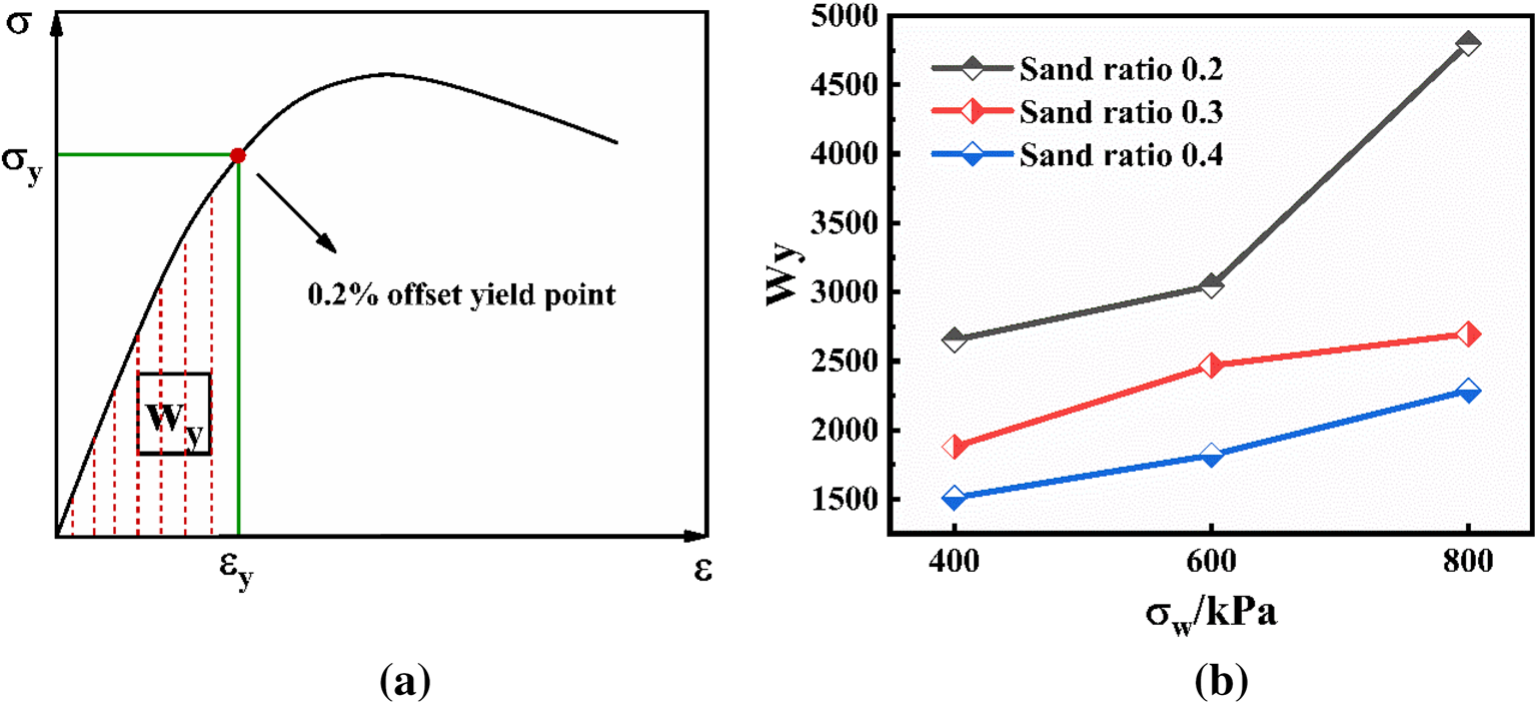
(**a**) Energy dissipation calculation diagram. (**b**) Relationship between dissipation energy and lateral confining pressure.

In order to analyze the energy dissipation when the CSG was irreversibly damaged under triaxial compression, the dissipated energy Wy when the specimen reached the yield point under different mix ratio and confining pressure was calculated by the method shown in Fig. 9a. The drawing took fly ash 40 kg/m^3^ and water-binder ratio 1.0 as an example, as shown in Fig. 9b. As could be seen from Fig. 9b, energy dissipation gradually increased with the increase of confining pressure value, but gradually decreased with the increase of sand rate. According to the calculated results, with the increase of confining pressure value, the constraint effect was continuously enhanced, so the dissipated energy of each specimen showed a gradual upward trend. In this process, as the CSG material was composed of cementitious material, sand and gravel and water, and the size of the sand and gravel particles was different, the strength of the sand and gravel itself was very high, coupled with the cementitious effect between the particles and the cementitious material, the initial damage of the material in the process of applying confining pressure was mainly composed of the bonding force between the cementitious material and the particles, the biting force between the particles and the friction resistance. Because the aggregate of CSG was strong, and the strength of cementitious material was relatively low, the initial damage was greater in the process of confining pressure, and irreversible damage was more likely to be formed in the process of stress. Therefore, the larger the sand ratio was, the lower the energy dissipation was.

### Damage analysis

In order to reflect the damage condition of CSG under triaxial compression, damage variable D was used to represent the damage condition of the specimen, 0 ≤ D ≤ 1, the larger the value of D, the higher the degree of damage of the specimen. The D value was calculated using the following formula.

2 $$D = 1 - \frac{{E^{*} }}{{E_{O} }}$$

where E^*^ is secant modulus; *E*_*0*_ is the secant modulus at 0.4 times the peak stress in the rising section of stress–strain curve.

**Figure 10:**
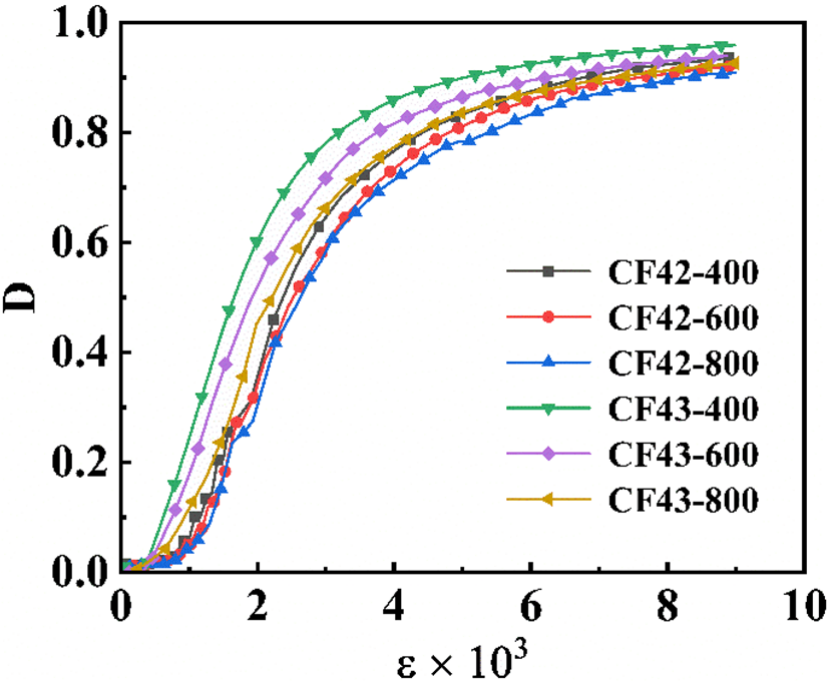
Relationship curves between strain and damage.

In order to clearly represent the damage changes under pressure in the figure, a curve and adjacent curves were randomly selected for plotting, and the results were shown in Fig. 10. As could be seen from Fig. 10, at the initial loading stage, the specimen was in the linear elastic stage without damage, and the damage variable D was close to 0. With the continuous increase of strain, each specimen appeared damage one after another. Among them, the damage development rate of CF42 was the highest when the confining pressure was 400 kPa, and the curve in the early stage showed a straight rise, while the curve slope of other compression specimens was relatively slow. With the increase of confining pressure, the enhancement of circumferential confinement inhibited the formation and development of cracks inside the specimen, and slowed down the damage evolution rate inside the specimen. When the damage curve entered the rapid development stage, the strain increased and the damage growth rate decreased. Compared with CF43, CF42 had lower initial damage stress and faster damage development rate. This was due to the difference between the internal crack evolution law of the two. When the crack developed from the cementing material to the coarse aggregate, its development direction would not change greatly, and the damage development would be slowed down. In addition, under the same cement content, fly ash content and confining pressure, the damage variable of sand rate 0.4 was greater than sand rate 0.3, which was consistent with the law mentioned above.

## Triaxial stress–strain curve prediction

### Methodology

#### XGBoost algorithm

**Figure 11:**
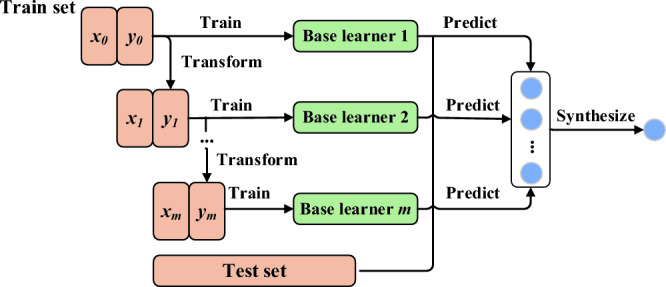
Boosting algorithm schematic diagram.

eXtreme Gradient Boosting (XGBoost) is also called the eXtreme Gradient Boosting tree. XGBoost algorithm is an integrated learning algorithm proposed by Chen et al.^53^ in 2014, aiming to make its application faster and more efficient. It is an algorithm realized on the basis of Gradient Boosting, which is shown in Fig. 11.

XGBoost, which includes Gradient Boosting Tree (GBDT), excels at accurately solving problems across a wide range of datasets. GBDT is an additive model based on boosting enhancement strategy. During training, the model is greedy-learned through the ante-term distribution algorithm. In each iteration, a CART tree is learned to fit the residual between the predicted value and the actual value of the previous tree. In general, XGBoost is a new model optimized based on GBDT, which has greatly improved both accuracy and stability. XGBoost model belongs to a tree ensemble model. Its core is to take the sum of the predicted values of each tree in all trees (assumed to be *K*) as the predicted value of this sample in the XGBoost model. Its function is expressed as follows:

3 $$\hat{y}_{i} = \phi \left( {{\mathbf{x}}_{i} } \right) = \sum\limits_{k = 1}^{K} {f_{k} \left( {{\mathbf{x}}_{i} } \right),\quad f_{k} \in {\mathcal{F}}}$$

Assuming that the data set has n samples and m features, it is defined as:

4 $$\mathcal{D}=\left\{\left({\mathbf{x}}_{i},{y}_{i}\right)\right\}\left(|\mathcal{D}|=n,{\mathbf{x}}_{i}\in {R}^{m},{y}_{i}\in R\right)$$

where *X*_*i*_ represents the ith sample, ${y}_{i}$ represents the label of the ith sample.

The space of the CART tree is F, and the formula is as follows:

5 $$\mathcal{F}=\left\{f(\mathbf{x})={w}_{q(\mathbf{x})}\right\}\left(q:{R}^{m}\to T,w\in {R}^{T}\right)$$

where *q* represents the tree model; *W*_*q(x)*_ represents the score set of all leaf nodes in *q*; *T* is the number of leaves in *q*.

The predicted value of the XGBoost model is the sum of the corresponding leaf node scores of all trees, that is, the sum of the predicted values of the corresponding samples of each tree. The goal of model machine learning is to learn all such tree models (assuming *f*(*x*)). In order to successfully learn model *f*(*x*), the following objective functions should be determined:

6 $${\mathcal{L}}\left( \phi \right) = \sum\limits_{i = 1}^{n} {l\left( {\hat{y}_{i} ,y_{i} } \right) + \sum\limits_{k = 1}^{K} {{\Omega }\left( {f_{k} } \right)} }$$

7 $$\Omega (f)=\gamma T+\frac{1}{2}\lambda \parallel {\text{w}}{\parallel }^{2}$$

where $\sum_{i = 1}^{n} l\left( {\hat{y}_{i} ,y_{i} } \right)$ is the loss function term, namely the training error; ${\sum }_{k=1}^{K}\Omega \left({f}_{k}\right)$ is the sum of the complexity of all trees, which can effectively prevent overfitting; $\hat{y}_{i}$ represents the predicted value of the model; *y*_*i*_ represents the label of the ith sample; *f*_*k*_ represents the kth tree model; *T* represents the number of leaf nodes per tree; *w* represents the set of the fractions of leaf nodes of each tree, $\gamma$ and $\lambda$ represent coefficients.

#### Cuckoo search algorithm

One of the effective methods to solve the optimal hyperparameters is grid search. With the increase of hyperparameter dimension, the computing cost of grid search increases exponentially. To mitigate this high computational burden, simulation algorithms like CS have emerged to optimize black-box functions more efficiently. Simulating the CS by searching for a bird's nest, the bird's nest and eggs can be considered as solutions, with constraints based on three rules: each cuckoo lays only one egg at a time, and randomly selects one nest to be placed; A randomly selected group of bird nests, with the best parasitic nest (i.e. solution) retained until the next generation; The number of bird nests is fixed, and the probability of the host discovering cuckoo eggs is Pa. If the host discovers bird eggs, they will destroy the eggs or search for new nests. The cuckoo bird's nest search path uses a global random walk based on Levy flight. Using Levy Flight to update the global optimal solution:

8 $${\Psi }_{l+1}^{i}={\Psi }_{l}^{i}+\alpha \otimes s$$

where α is the scale of the step size, take α = 1; $s=\frac{u}{{\left|v\right|}^{1/\omega }}\sim l{e}^{\prime}vy\left(\omega \right)$, $u\sim N\left(0,{\sigma }_{l}^{2}\right)$, *v* ~ *N*(0,1), $\omega$=1.5

#### CS-XGBoost model

The predictive performance of the XGBoost model hinges on the parameter settings. Leveraging the CS algorithm to optimize XGBoost parameters can enhance model effectiveness. Here are the key steps to construct the CS-XGBoost model:

Step 1 The corresponding parameters of CS algorithm are determined, the maximum number of elections *L*, the nest discovery probability *Pa*, and the initial solution ${\Psi }_{0}=(\theta , \eta , m, n)$_;_

Step 2 Initialize the XGBoost parameters ${\Psi }_{1}{,\Psi }_{2}{, \dots ,\Psi }_{d}$, define the objective function $\varphi \left({\Psi }_{i}\right), i=\mathrm{1,2},\dots ,d$ as the loss function value of the XGBoost model test set, *d* is defined as the number of solutions;

Step 3 *d* solutions choose one solution at random *Ψ*_*i*_*,* calculate the objective function $\varphi \left({\Psi }_{i}\right)$, the nest location is obtained by using formula (8) update, calculate the objective function $\varphi \left({\Psi }_{j}\right)$, if $\varphi \left({\Psi }_{i}\right)$>$\varphi \left({\Psi }_{j}\right)$, then new solution $\varphi \left({\Psi }_{j}\right)$ replaces $\varphi \left({\Psi }_{i}\right)$.

Step 4 Traverse ${\Psi }_{i}$ through the objective function $\varphi \left(\right)$,$i=\mathrm{1,2},\dots ,d,$ to find the optimal parametric solution ${\Psi }_{l}^{opt}$, keep ${\Psi }_{l}^{opt}$ to the next iteration, discard other non-optimal parametric solutions with probability *Pa*, and search for new solutions through Levy flight;

Step 5 The iteration terminates with ${\Psi }_{l}^{opt}$, and the calculation is carried into the XGBoost model.

### Data analysis and preprocessing

#### Data analysis

**Figure 12:**
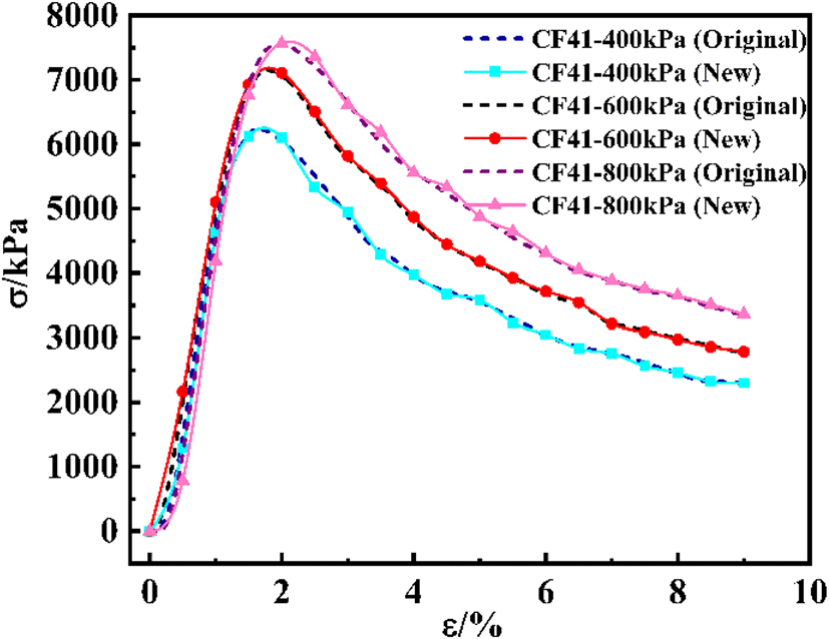
Comparison between the curve composed of 18 feature points and the original curve.

Diverging from conventional data prediction approaches, this paper expected to realize the prediction of the entire stress–strain curve. Referring to Zheng^54^, before using machine learning to predict the triaxial experimental constitutive curve of sand, the actual measured curve was divided into multiple points to realize the transformation of curve prediction to multiple feature points. The strain value of the curve obtained in this paper reached 9% in a unified way. The difference was that Zheng's data was sourced from other literature and digitized images with Enguage Digitizer, while the data in this paper came from experiments with a detailed curve-building process. In this paper, we tried to take points of the curve according to 0.5% strain value interval and found the corresponding stress value. A total of 18 stress values were selected as the output characteristics of the model. Consistent with the test mix ratio, as the cement content was uniformly 50 kg/m^3^, there were 4 input variables in the model, namely fly ash content, water-binder ratio, sand rate and confining pressure, and 18 output variables, which were 18 stress values obtained successively on the curve. In order to verify the feasibility of taking points successively as output features according to the 0.5% strain value, a set of mix ratio was selected and the curve composed of 18 points divided was compared with the original test curve, as shown in Fig. 12. It could be seen that the curve composed of 18 points obtained was highly consistent with the original test curve, and this method was feasible.

#### Data preprocessing

Due to the differences in the units of the input variables in this study, if they are directly substituted into the model for learning, it will have a certain impact on the final results. It is necessary to normalize the data. The normalization process retains all the features, converts the parameter values into data between 0 and 1, and transforms the dimensional expressions into non-dimensional expressions so that the data are numerically comparable and the model is more accurate and stable. In this study, min–max normalization was used as the normalization method, and the expression formula was as follows:

9 $${y}_{i}=\frac{{x}_{i}-{x}_{{\text{min}}}}{{x}_{{\text{max}}}-{x}_{{\text{min}}}}$$

where *y*_*i*_ represents the normalized data, *x*_*min*_ represents the minimum feature data; *x*_*max*_ indicates the maximum value of the feature. *x* is the data before normalization.

### Model performance analysis

The data set was divided into training set and test set according to 8:2. After the data was preprocessed, the XGBoost prediction model based on the initial parameters was established through the training set. The CS algorithm was used to optimize the six hyperparameters of the XGBoost model (learning rate, n_estimators, max_depth, min_child_weight, subsample, colsample_bytree), and the number of iterations of the CS hyperparameters $\Psi =(\theta , \eta , m, n)$ was set to 50. After optimization, the hyperparameters were substituted into the XGBoost model, and the CS-XGBoost model was generated by retraining, and the model was verified by using the test set for prediction. To analyze the prediction effect of the model, the evaluation indicators included Coefficient of Determination (R^2^), Root Mean Squared Error (RMSE) and Mean Absolute Error (MAE). The formula was as follows:

10 $${R}^{2}=1-\frac{\sum_{z=1}^{N}{\left({y}_{z}-{f}_{z}\right)}^{2}}{\sum_{z=1}^{N}{\left({y}_{z}-\overline{{y }_{z}}\right)}^{2}}$$

11 $$RMSE=\sqrt{\frac{\sum_{i=1}^{N}{\left({y}_{i}-{f}_{i}\right)}^{2}}{N}}$$

12 $$MAE=\frac{\sum_{i=1}^{N}\left|{y}_{i}-{f}_{i}\right|}{N}$$

where *N* is the number of sample data; *y*_*i*_ is the experimental value, MPa ; $\bar{y}_{i}$ is the average of the experimental value, MPa; *f*_*i*_ is the predicted value of model regression, MPa; $\bar{f}_{i}$ is the average of the predicted value from the model regression, MPa.

After the CS algorithm optimized the hyperparameters, the model achieved the optimal accuracy. After the test set verification, the average R^2^ value of 18 features in the XGBoost model based on initial parameters was 0.8573, while the average R^2^ value of 18 features in the CS-XGBoost model could reach 0.9516. The specific values of different feature indexes in the CS-XGBoost model were shown in Tables 4 and 5. It could be seen that the accuracy of different features was similar, relatively uniform, and could reach a high level at the same time. In the training set, the lowest R^2^ was 0.9528, the highest was 0.9824, and in the test set, the lowest R^2^ was 0.9305, the highest was 0.9640. Meanwhile, RMSE and MAE were in a small range. In addition, no matter the index of a single feature or the overall average, R^2^, RMSE and MAE of the training set and the test set were not much different, which indicated that the CS-XGBoost model had high prediction accuracy while there was no overfitting or underfitting phenomenon, and the model was feasible.

**Table 4:** CS-XGBoost model prediction results of different characteristics index statistics: Train set.

Train set	y_1_	y_2_	y_3_	y_4_	y_5_	y_6_	y_7_	y_8_	y_9_
	y_10_	y_11_	y_12_	y_13_	y_14_	y_15_	y_16_	y_17_	y_18_
R^2^	0.9528	0.9566	0.9624	0.9669	0.9673	0.9797	0.9824	0.9789	0.9767
	0.9796	0.9781	0.9772	0.9752	0.9745	0.9724	0.9709	0.9708	0.9713
RMSE	284.2090	455.7508	432.8228	471.3809	477.2624	389.3593	357.3507	360.5903	352.2550
	321.2507	315.2331	308.0210	304.7871	292.9908	290.4446	283.7100	275.4995	263.0004
MAE	237.9441	353.3664	312.1514	380.0501	395.3002	299.9150	280.7326	292.0006	285.4325
	252.1444	242.2301	235.3627	233.7447	228.2127	225.3445	226.4895	219.1687	213.1248

**Table 5:** CS-XGBoost model prediction results of different characteristics index statistics: Test set.

Test set	y_1_	y_2_	y_3_	y_4_	y_5_	y_6_	y_7_	y_8_	y_9_
	y_10_	y_11_	y_12_	y_13_	y_14_	y_15_	y_16_	y_17_	y_18_
R^2^	0.9305	0.9351	0.9423	0.9573	0.9443	0.9584	0.9640	0.9524	0.9560
	0.9557	0.9565	0.9513	0.9471	0.9629	0.9515	0.9561	0.9545	0.9531
RMSE	277.7442	378.0496	324.2293	354.7321	431.2674	475.8576	429.6305	442.2685	430.9649
	407.7955	377.6169	348.4335	345.9149	280.6147	316.0527	306.9779	287.6409	292.9544
MAE	223.8429	314.1321	274.2810	295.6529	346.5636	424.8335	371.5975	380.8595	378.7548
	371.5438	325.8899	284.3746	284.1389	230.6240	273.3044	238.5586	225.2943	250.9486

**Figure 13:**
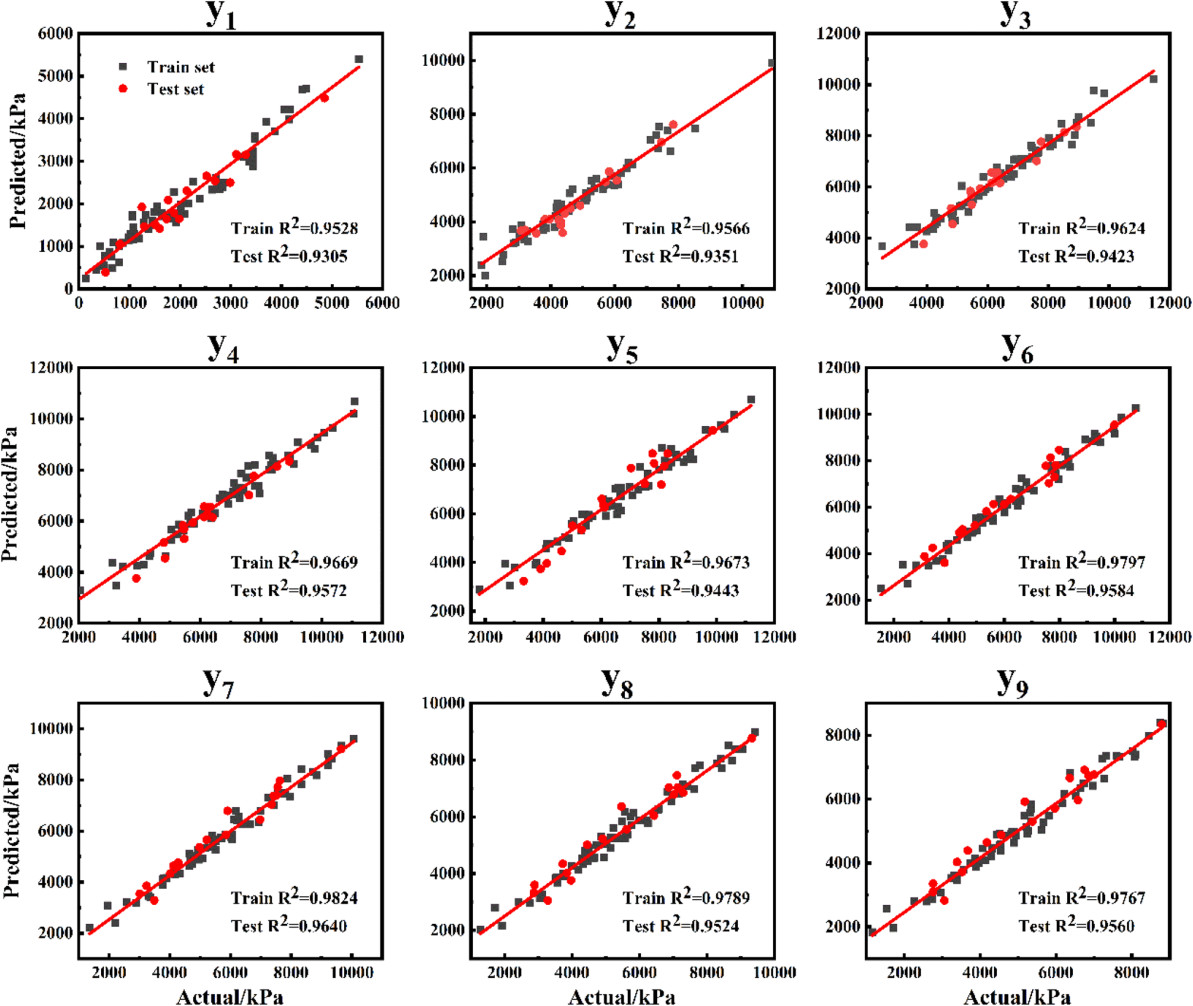
Linear fitting between the predicted values and the actual values of different features of the CS-XGBoost model: y_1_–y_9_.

**Figure 14:**
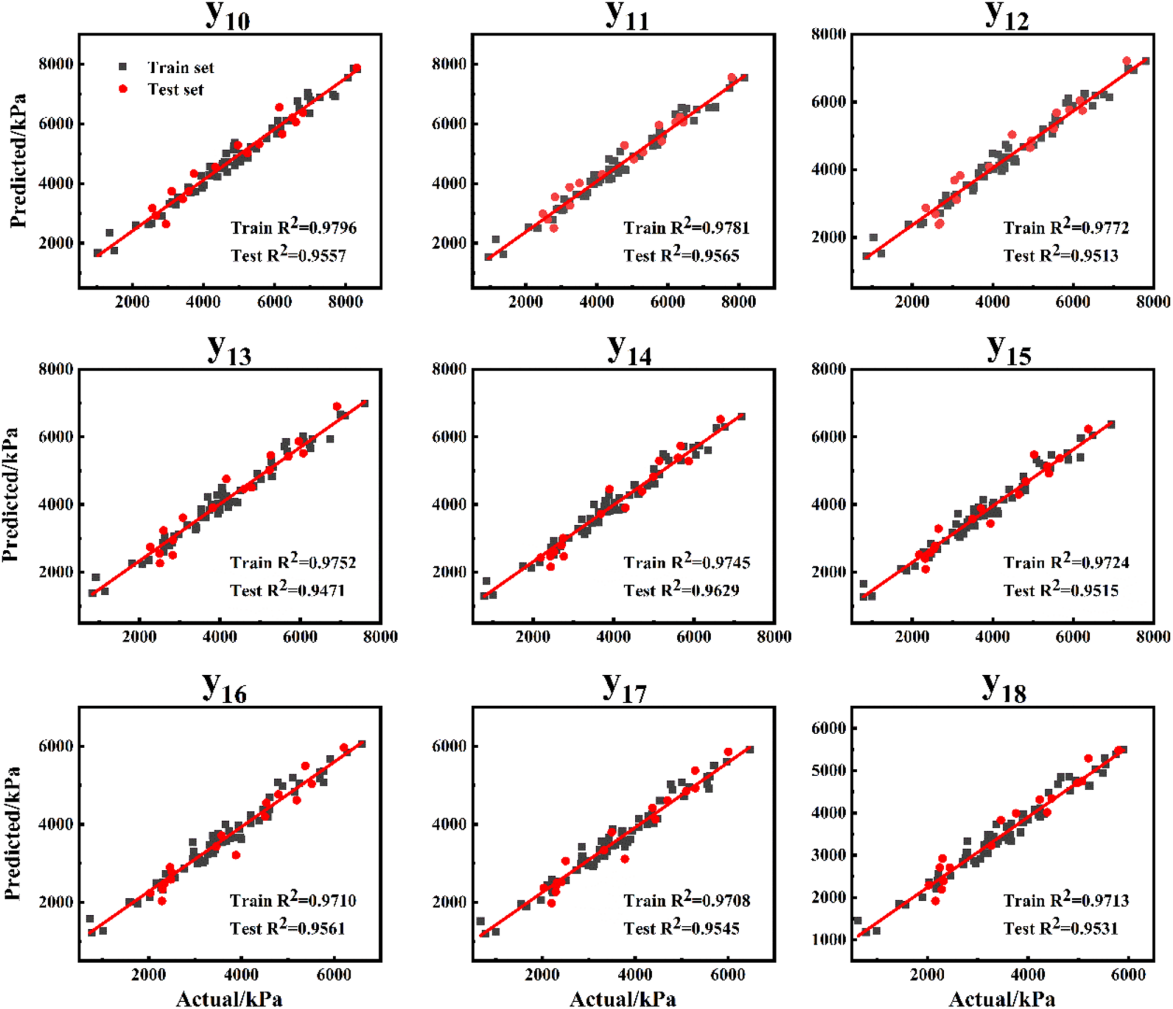
Linear fitting between the predicted values and the actual values of different features of the CS-XGBoost model: y_10_–y_18_.

To better visualize the prediction accuracy and performance of the model, a linear fitting diagram was created to display the relationship between the predicted results of the model and the actual values. The diagrams were shown in Figs. 13 and 14. After the CS algorithm optimized the hyperparameters, the model reached the optimal accuracy and became more stable. It could be seen from the fitting status of the model in the training set and the test set shown in Figs. 13 and 14 that the model performed well in both the training set and the test set. It showed that the predicted values of different characteristics of CS-XGBoost model were in excellent agreement with the actual values, and the model was feasible for the prediction of stress–strain curves.

**Figure 15:**
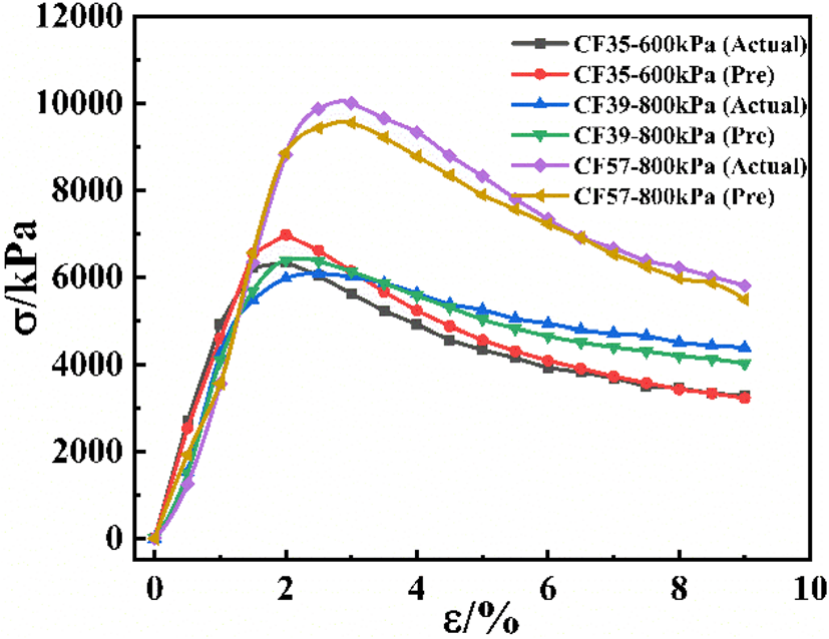
Comparison between the predicted curve of CS-XGBoost model and the actual curve.

**Figure 16:**
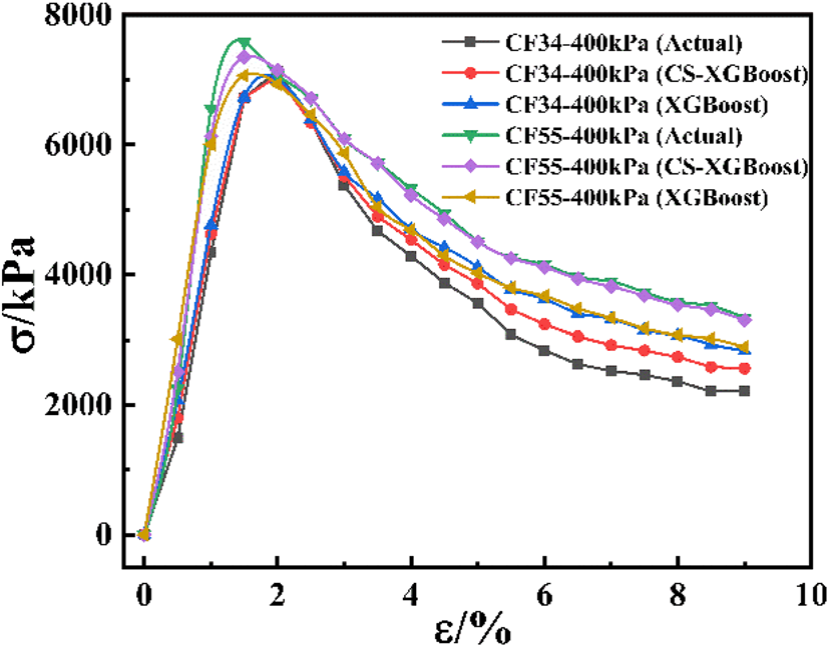
Comparison of prediction curves of different models.

The above chart illustrated the prediction performance of the model in different features and as a whole, but the prediction effect of the model on the stress–strain curve was not directly expressed. Therefore, three groups of data were randomly selected from the test set, and the prediction curve of the CS-XGBoost model was compared with the actual curve to observe the prediction effect of the model, as shown in Fig. 15. It could be seen from Fig. 15 that the predicted curves in the three groups of data had a high degree of fit with the actual curves, which further intuitively indicated that the CS-XGBoost model had a high prediction accuracy and could be applied to the prediction of the triaxial stress–strain curve of CSG. In addition, in order to directly represent the advantages of CS algorithm optimization of hyperparameters, two groups of data were randomly selected from the test set, and the actual curve was drawn and compared with the prediction curve of CS-XGBoost and XGBoost model, as shown in Fig. 16. It could be seen from Fig. 16 that in both groups of data, the prediction curve of CS-XGBoost model had better fit with the actual curve, while the error of XGBoost model was the largest, indicating that the CS-XGBoost model had better prediction accuracy and significantly improved its prediction performance. In conclusion, optimization of hyperparameters by CS algorithm had significant effectiveness in improving model performance.

### Discussion

Through the conventional triaxial test of CSG, the influence laws of different characteristics such as stress–strain curve and elastic modulus were analyzed, and the CS-XGBoost combined model was used to predict the stress–strain curve. The research results obtained were similar to those of previous studies. Yang et al.^10^ conducted triaxial tests on CSG with different cement content and found that the peak stress, peak strain and initial elastic modulus of CSG increased with the increase of confining pressure. Sun et al.^55^ conducted relevant performance test analysis on CSG and found that the “optimal sand rate” was about 20% in the common mix ratio range of engineering, which was consistent with the phenomenon that the peak stress–strain curve would decrease to a certain extent when the sand rate rose from 20 to 40% in this study; The study on the effect of fly ash content also found that the optimal content of fly ash was 50% of the total amount of cementing material, which was consistent with the phenomenon in this study that the peak value of stress–strain curve with 50% fly ash content was significantly higher than 30% and 40% when the cement content was 50 kg/m^3^; In addition, the influence of water-binder ratio was studied and it was found that the optimal water-binder ratio corresponding to the common sand rate of 0.1–0.4 in engineering was 1.0–1.4. When the sand rate was high, the corresponding optimal water-binder ratio was set at the upper limit, and vice versa. When the sand ratio was 0.2 and 0.3 in this study, the peak value of the stress–strain curve appears at the inflection point when the water-binder ratio was 1.2, which conforms to the above law. Chen et al.^56^ conducted a study on the triaxial mechanical properties of coral sea sand concrete, and concluded that with the increase of confining pressure, the energy dissipation of each specimen showed a gradual upward trend, while the damage development rate of the specimen gradually slowed down. Although different from the materials in this study, they also had similar rules as building materials. In summary, the conclusions drawn in this study had certain reliability.

Hu^57^ applied CS-XGBoost model to breast cancer diagnosis, and the results showed that CS-XGBoost model had the advantages of small error and high precision, and had a good performance in the accuracy, F1 score and other performance indicators. The cuckoo search algorithm could effectively train the model parameters and greatly improved the model performance. Before training and predicting the stress–strain curve of the concrete micro model through machine learning, Zhou et al.^58^ also preprocessed the curve by characterizing different points on the curve, and the model achieved excellent predictive performance. Yu et al.^59^ used XGBoost model to predict the dynamic frequency response curve of power system. The results showed that the model could successfully predict the curve and had great generalization ability. The parameter training method, curve processing method, prediction model and effect were similar to those in this study, indicating that the CS-XGBoost model proposed in this paper could be used as a reliable prediction method for CSG stress–strain curve.

## Conclusion

In this paper, a series of triaxial compression tests were carried out on CSG materials with different mix proportions under different confining pressures. Based on the experimental results, the stress–strain curve, elastic modulus, energy loss and damage analysis of CSG materials were analyzed. The conclusions are as follows:

1. Under the same cement content, sand ratio, fly ash content and confining pressure, an optimal water-to-binder ratio of 1.2 was observed for sand ratios of both 0.2 and 0.3. Sand rate had a notable impact on the stress–strain curve, and the peak stress and strain decreased with the increase of confining pressure. Under the same mix proportions, the peak strength of the CSG material specimen increased with the increase of confining pressure, and the two showed a relatively obvious linear correlation. When the CSG material reached the peak failure value, the corresponding strain value was about 2%, and the strain value also increased with the increase of confining pressure value.
2. The energy dissipation of all specimens increased with the increase of confining pressure value. Since the CSG materials were more likely to form irreversible damage during the stress process, the larger the sand rate, the lower the energy dissipation trend. At the initial stage of loading, the specimen was in the non-damaging linear elastic stage, and the damage variable D of the specimen was close to 0. With the increase of confining pressure, the damage evolution rate inside the specimen was slowed down due to the circumferential constraint.
3. In this paper, the triaxial stress–strain curve data of 81 groups of tests were randomly divided into 64 groups of training set data and 17 groups of test set data after processing. The average coefficient of determination of the proposed CS-XGBoost model under 18 different output characteristics reached 0.9516, and the prediction curve had a high consistency with the test curve. The results showed that this model could predict the triaxial stress–strain curve of CSG quickly and accurately.
4. By comparing the predicted results of the CS-XGBoost model and the XGBoost model, the average value of the performance index R^2^ of the CS-XGBoost model was increased by 10.10% compared with the XGBoost model with initial parameters, indicating that parameter optimization by CS algorithm could significantly improve the performance based on the initial parameter model and had obvious advantages.

In this paper, the conventional triaxial test of CSG was conducted to analyze the influence laws of stress–strain curve, specimen failure form, elastic modulus, energy dissipation and damage evolution, and corresponding results and laws were obtained. At the same time, CS-XGBoost model was proposed to predict the triaxial stress–strain curve of CSG, which successfully predicted and achieved high prediction accuracy. However, there were some limitations. It can be found that some phenomena in the chart in this paper are outside the rule, which may be due to the influence of experimental equipment or samples, but it dose not affect the overall rule statistics. It is suggested to further enrich the conventional triaxial test of CSG in future studies. In addition, it is very important to evaluate the generalization ability of the model, which is also one of the challenges facing the current research field. For future studies, an approach worth considering is utilizing more relevant data to assess the generalization capacity of the model introduced in this paper.

## Data Availability

The data that support the findings of this study are available from the corresponding author upon reasonable request.
